# Familial *Trichostrongylus* Infection Misdiagnosed as Acute Fascioliasis

**DOI:** 10.3201/eid2110.141392

**Published:** 2015-10

**Authors:** Keyhan Ashrafi, Ali Tahbaz, Meysam Sharifdini, Santiago Mas-Coma

**Affiliations:** Author affiliations: Guilan University of Medical Sciences, Rasht, Iran (K. Ashrafi, M. Sharifdini);; Rasoul-Akram Hospital, Rasht (A. Tahbaz);; Universidad de Valencia, Burjassot, Spain (S. Mas-Coma)

**Keywords:** *Trichostrongylus*, fascioliasis, misdiagnosis, parasites, zoonoses, Iran

**To the Editor:** Human fascioliasis, infection with *Fasciola* spp. flukes, is highly pathogenic in both acute and chronic phases and can result in death (*1*). This disease has been recently emerging, in part linked to climate and global changes (*2*). Human *Fasciola* infection has been reported in 5 continents and is related to the disease’s wide spread in livestock. Guilan Province in northern Iran is a fascioliasis-endemic area where the largest human epidemics have occurred, together affecting ≈15,000 persons (*3*).

In 2014, 3 sisters (ages 35, 33, and 38) and their 41-year-old brother (patients 1–4, respectively) sought medical care at the same time, all with a 3-week history of symptoms. The patients lived in the Langroud district in Guilan Province, at the Caspian Sea littoral; the sisters lived in the same household with their parents, and the brother lived in a nearby household with his wife and son. Patient 1 had mild abdominal and epigastric pain radiating to her back; onset of abdominal pain and flushing during meals; rigors most prominent at night; severe and voluminous diarrhea intensifying after meals; poor appetite; and urticaria associated with itching on her back, chest, and neck. Patient 2 had epigastric and severe right upper quadrant pain that radiated to her back; severe liver tenderness; weakness; nausea; flatulence; continuous diarrhea; urticarial lesions associated with itching on hands, abdomen, and chest; and a history of backache and pulmonary allergy a few months earlier. Patient 3 had abdominal and epigastric pain, loose defecation, and flatulence; urticaria on the neck; and flushing. Patient 4 had abdominal, neck and shoulder pain; constipation; dyspepsia; severe flatulence; and low-grade fever.

Eosinophil levels for patients 1–4, respectively, were 16,260, 2,640, 13,104, and 3,523 cells/mm^3^. Results from liver function tests were within reference ranges except for lightly increased alanine aminotransferase (35 IU/L) for patient 2. Sonography of liver, pancreas, and spleen showed no abnormalities. Serologic test results for antibodies to *Fasciola* and *Strongyloides* were negative. All patients denied close contact with herbivorous animals but mentioned regular consumption of fresh vegetables from local markets or from the parents’ home garden; the latter had been fertilized with sheep manure a few months before symptom onset.

Acute fascioliasis was diagnosed on the basis of symptoms, weight loss, hypereosinophilia, vegetable consumption, and residence in a high-risk area, all typical associations with this illness (*1*). Absence of fasciolid eggs in stools by Kato-Katz and formalin-ether coprologic methods and lack of sonographic abnormalities were explained by the illness’s early invasive phase, and negative serologic results were explained by the immunologic response heterogeneity in fascioliasis (*4*) or antigen deterioration. The patients were treated with a single dose of triclabendazole (10 mg/kg).

One month after treatment, patients returned with the same symptoms and hypereosinophilia. Unexpectedly, patients 1–3 showed *Trichostrongylus* eggs in stools (Figure, panel A). The fourth patient’s stool sample was negative. Samples from other family members were analyzed, and 4 (patients’ father and mother and the man’s wife and son) were shedding *Trichostrongylus* eggs, for a total of 7 patients shedding eggs. *T. colubriformis* and *T. vitrinus* nematodes (Figure, panels B and C) were identified in feces 24 hours after treatment. This diagnosis was surprising because, although prevalences as high as 71% for *Trichostrongylus* spp. in humans have been described in central and southern Iran (*5*), only sporadic cases have been recently reported.

**Figure:**
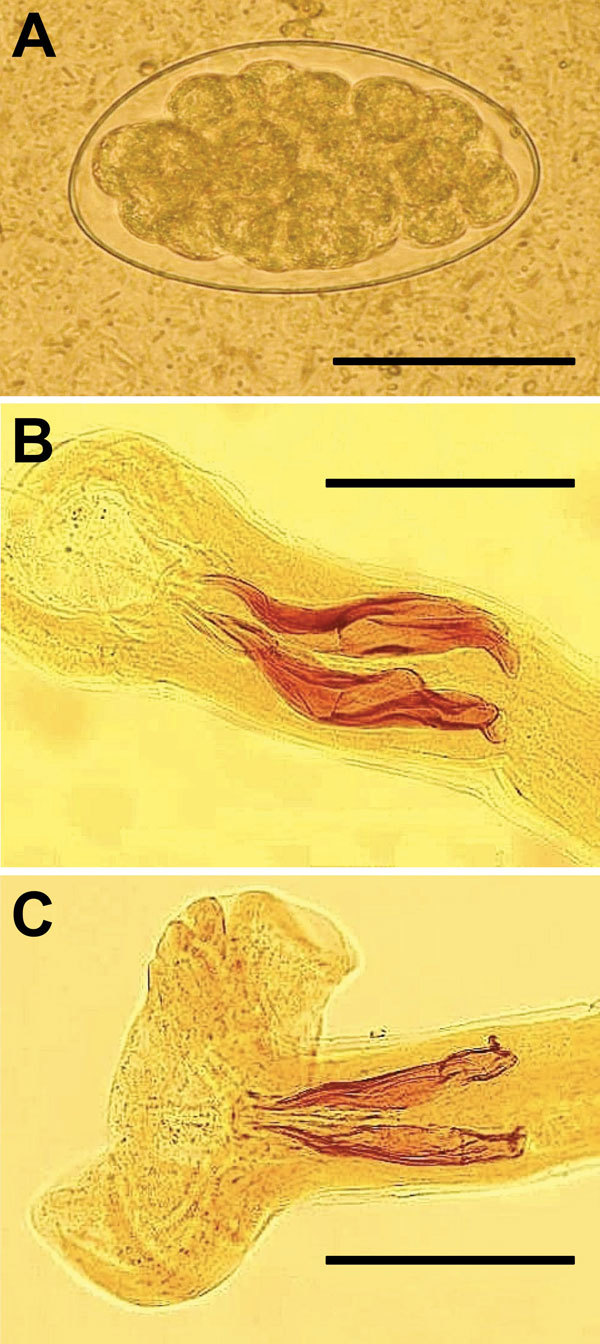
*Trichostrongylus* spp. eggs and nematodes isolated from 1 patient in Guilan Province, Iran, 2014. A) Egg of diameter 87.5 × 48.0 μm obtained from fecal sample of patient by using formalin-ether method. Scale bar indicates 50 μm. B) Bursa copulatrix and spicules (slightly unequal, 135–156 µm long, and boat-shaped with a stepped tip and an outgrowth capping at proximal end) of *T. colubriformis* adult male. Scale bar indicates 100 μm. C) Bursa copulatrix and spicules (equal in size, 160–170 µm long, and straight and tapering sharply at distal end) of *T. vitrinus* adult male. Scale bar indicates 100 μm.

Infection intensity (24–300 eggs per gram [epg] of feces) correlated with clinical manifestations, indicating light (10–99 epg) to moderate (100–999 epg) severity (*6*). The patient with 24 epg was asymptomatic, and light or moderate cases are known to be asymptomatic (*5*). The acute symptomatology in these patients might be explained by their emaciated and weak conditions. Absence of eggs in the initial analyses may be explained by the long prepatent period (4 months–2 years) and by egg shedding discontinuity in light or moderate infections (*6**–**8*).

The patients fully recovered, and their eosinophilia returned to reference values <1 month after treatment with 1 dose of albendazole (400 mg), followed by mebendazole (200 mg/day for 3 days). One patient who only partially responded was successfully retreated 1 month later.

Trichostrongyliasis and fascioliasis share many epidemiologic and clinical characteristics. *Trichostrongylus* spp. infect livestock worldwide, and human infection has been reported in many countries. Eggs are excreted with feces and then hatch and develop into strongyloid larvae. Humans become infected when ingesting these larvae along with vegetables contaminated by animal feces (*9*). Climate change has been suggested as contributing to the increasing risk for human infection by *Trichostrongylus* spp. (*10*). Trichostrongyliasis patients have symptoms like those reported here, although mild eosinophilia may sometimes be the only indication (*6**–**8*). Familial outbreaks related to consumption of fresh vegetables fertilized with sheep and goat manure have been reported (*6**–**9*).

This familial infection cluster highlights the need to consider trichostrongyliasis in patients with suspected fascioliasis in acute or chronic phases without eggs in stools. This diagnosis is especially possible if patients have consumed fresh vegetables fertilized with fresh livestock manure or have had close contact with herbivorous animals.
